# iVUN: interactive Visualization of Uncertain biochemical reaction Networks

**DOI:** 10.1186/1471-2105-14-S19-S2

**Published:** 2013-11-12

**Authors:** Corinna Vehlow, Jan Hasenauer, Andrei Kramer, Andreas Raue, Sabine Hug, Jens Timmer, Nicole Radde, Fabian J Theis, Daniel Weiskopf

**Affiliations:** 1 Visualization Research Center (VISUS), University of Stuttgart, Allmandring 19, 70569 Stuttgart, Germany; 2 Institute of Bioinformatics and Systems Biology, Helmholtz Zentrum München, Ingolstädter Landstraße 1, 85764 Neuherberg, Germany; 3 Department of Mathematics, Technische Universität München, Boltzmannstraße 3, 85748 Garching, Germany; 4 Institute for Systems Theory and Automatic Control, University of Stuttgart, Pfaffenwaldring 9, 70550 Stuttgart, Germany; 5 Institute for Physics, University of Freiburg, Hermann-Herder Straße 3, 79104 Freiburg, Germany; 6 Freiburg Institute for Advanced Studies (FRIAS), University of Freiburg, Albertstraße 19, 79104 Freiburg, Germany; 7 BIOSS Centre for Biological Signalling Studies, University of Freiburg, Schänzlestraße 18, 79104 Freiburg, Germany

## Abstract

**Background:**

Mathematical models are nowadays widely used to describe biochemical reaction networks. One of the main reasons for this is that models facilitate the integration of a multitude of different data and data types using parameter estimation. Thereby, models allow for a holistic understanding of biological processes. However, due to measurement noise and the limited amount of data, uncertainties in the model parameters should be considered when conclusions are drawn from estimated model attributes, such as reaction fluxes or transient dynamics of biological species.

**Methods and results:**

We developed the visual analytics system *iVUN *that supports uncertainty-aware analysis of static and dynamic attributes of biochemical reaction networks modeled by ordinary differential equations. The multivariate graph of the network is visualized as a node-link diagram, and statistics of the attributes are mapped to the color of nodes and links of the graph. In addition, the graph view is linked with several views, such as line plots, scatter plots, and correlation matrices, to support locating uncertainties and the analysis of their time dependencies. As demonstration, we use *iVUN *to quantitatively analyze the dynamics of a model for Epo-induced JAK2/STAT5 signaling.

**Conclusion:**

Our case study showed that *iVUN *can be used to perform an in-depth study of biochemical reaction networks, including attribute uncertainties, correlations between these attributes and their uncertainties as well as the attribute dynamics. In particular, the linking of different visualization options turned out to be highly beneficial for the complex analysis tasks that come with the biological systems as presented here.

## Background

Biomolecules, such as genes, RNAs and proteins, are the building blocks of cells. Via different modes of interaction, these biomolecules (also called biochemical species) form gene regulatory, signaling and metabolic pathways. In systems biology, biochemical reaction network (BRN) models are used to describe the structure of these pathways and the interaction between biochemical species [1,2]. As BRN models can be employed to summarize all available information, they are a powerful tool that can be used to gain a holistic understanding of cellular processes and their crosstalk.

A variety of approaches are available to model BRNs. In particular ordinary differential equations (ODEs) are widely used. ODE models allow for the description of the time evolution of the concentrations of the chemical species based upon knowledge of the reactions, their rates constants and other parameters. While the set of possible reactions is often known, the parameters can in general not be measured. To ensure reliability and predictive power of the BRN models, the unknown parameters have to be estimated from the available measurement data. Due to the limited availability of data and the ubiquity of measurement noise, the parameter estimation does in general not yield a unambiguous result, i.e., the parameters remain uncertain. To analyze the uncertainty of the parameters as well as the model prediction, often a sample of parameters is collected for which model simulations and data agree reasonably well [3-5]. To draw grounded conclusions about the systems' behavior, the uncertainties encoded in this sample have to be studied. Although there are various tools available that help simulating and visualizing BRN models, hardly any tool exists that supports the visual analysis of uncertainties in BRN models.

In the following, we present our visual analytics system *iVUN*. *iVUN *supports an in-depth study of BRNs with uncertain properties. We compute the uncertainties of parameters and model predictions using a Bayesian estimation approach, which provides the statistics of model attributes (parameters, reaction fluxes and states) in form of a sample. Given this sample, *iVUN *facilitates the study of attribute uncertainties and their time-dependence by visualizing them in the graph view, a node-link diagram. Biochemical species and reaction related attributes are mapped to the color of the nodes and links, respectively. Furthermore, a multitude of linked views, such as line plots, scatter plots, and correlation matrices, are available to enable the user to explore the BRN model and its uncertainties. We note that this manuscript is based on a conference paper we published at the 2nd IEEE Symposium on Biological Data Visualization [6] and uses original material thereof. In contrast to this visualization paper, here, we focus on the application to biological data obtained from experiments and included an extensive case study. Furthermore, *iVUN *has been extended to analyze the dynamics of measured quantities of the BRNs, which are neither states nor fluxes, within one and across different experimental conditions. This allows for the direct comparison of model predictions and measurement data, as well as the comparison of different experimental conditions. To improve the visual analytics capabilities of *iVUN*, we also introduced additional links between the available views.

**Figure 1:**
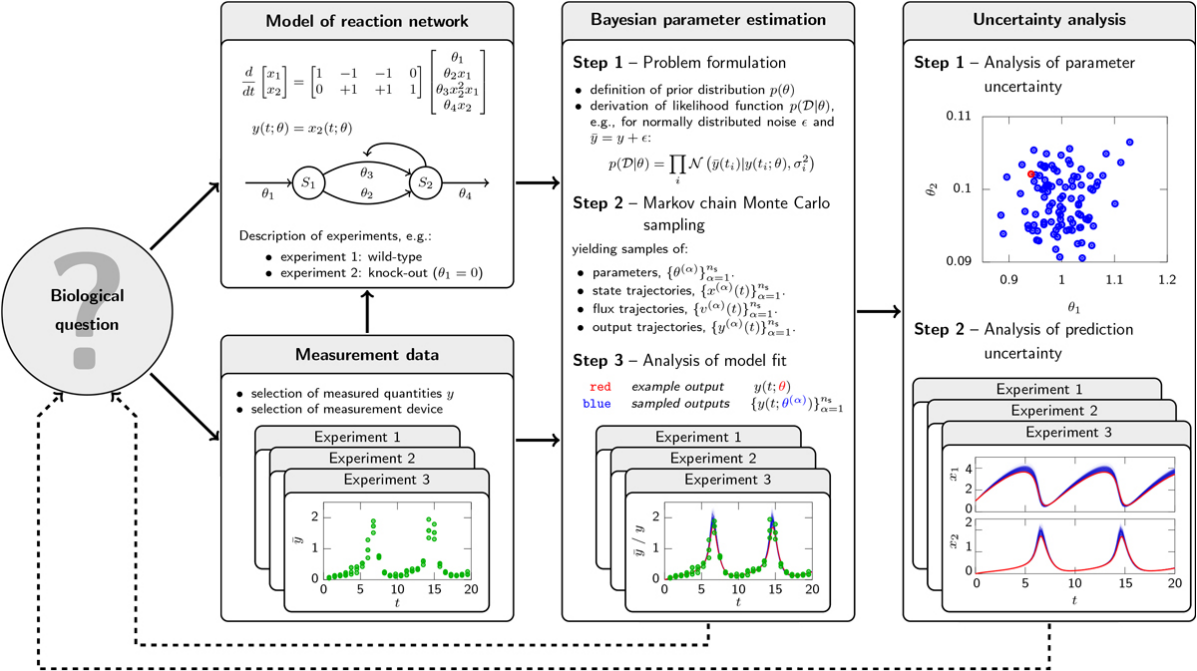
**Workflow of model development**. Answering a biological question using data-driven mechanistic modeling requires at least four essential steps: collection of measurement data (left, bottom); derivation of ODE model (left, top); estimation of the parameters of the ODE model using the measurement data (middle); and analysis of the fitted model, including the parameter and prediction uncertainties (right). Depending on the complexity of the problem, theses steps have to be iterated.

In the next section, we provide a brief review on ODE-based modeling approaches for BRNs. Furthermore, we introduce Bayesian parameter estimation and uncertainty analysis methods for ODEs (see Figure 1). Based upon these backgrounds, we define the aims of the analysis and hence visualization requirements.

### Computational modeling of biochemical reaction networks

#### Biochemical reaction networks

BRNs are defined by sets of biochemical species $\left(X_{\text{1}},X_{\text{2}},\ldots ,X_{n_{\text{x}}}\right)$ and biochemical reactions $\left(R_{\text{1}},R_{\text{2}},\ldots ,R_{n_{\text{r}}}\right)$. Biochemical species are ensembles of chemically identical molecular entities, e.g., RNAs and proteins [7]. Reactions are processes which result in the interconversion of some biochemical species (reactants, r) in some others (products, p), and can be written as:

$$R_{j}:\;\overset{n_{x}}{\underset{i=1}{\sum }}s_{ij}^{\left(\text{r}\right)}X_{i}\to \overset{n_{x}}{\underset{i=1}{\sum }}s_{ij}^{\left(\text{p}\right)}X_{i},\;j=1,\ldots ,n_{\text{r}}.$$

Here, $s_{ij}^{\left(\text{r}\right)}\in {\mathbb{N}}_{0}$ and $s_{ij}^{\left(\text{p}\right)}\in {\mathbb{N}}_{0}$ are the stoichiometric coefficients of species *i *in reaction *j*, which denote the number of molecules consumed and produced when the reaction takes place [1], respectively. The overall reaction stoichiometry is

$$S_{ij}=s_{ij}^{\left(\text{p}\right)}-s_{ij}^{\left(\text{r}\right)}\;\text{for }i=1,\ldots ,n_{\text{x}},\;\text{and }j=1,\ldots ,n_{\text{r}},$$

in which $S=\left\{S_{ij}\right\}\in {\mathbb{Z}}^{n_{\text{x}}\times n_{\text{r}}}$.

The species and reactions of a BRN can be interpreted as vertices and edges of a graph. This graph contains two types of edges: regular directed edges representing the interconversions between species (these vertices are encoded in the stoichiometric matrix *S*); and directed hyper-edges from a species to a reaction. The hyper-edges describe dependencies of the reaction rates on biochemical species (often called modifiers) which are not consumed by the reaction.

#### Ordinary differential equation models of BRNs

The dynamics of BRNs can be described using many different approaches. In this manuscript we considered ODE models of BRNs which are commonly written as:

(1) $$\dot{x}=Sv(x,\theta ,u),\;x(0)=x_{0}(\theta ,u),$$

in which $x\left(t\right)\in {\mathbb{R}}_{+}^{n_{\text{x}}}$ is the state at time *t*, with *x_i _*being the concentration of the chemical species *X_i_*. Furthermore, $x_{0}\left(\theta ,u\right)\in {\mathbb{R}}_{+}^{n_{\text{x}}}$ is the parameter and experiment dependent initial condition, $S\in {\mathbb{Z}}^{n_{x}\times n_{\text{r}}}$ is the stoichiometric matrix, $v\left(x,\theta ,u\right)\in {\mathbb{R}}_{+}^{n_{\text{r}}}$ is the flux vector, and $\theta \in {\mathbb{R}}_{+}^{n_{\theta }}$ is the parameter vector. The potentially time-dependent function $u\left(t\right)\in {\mathbb{R}}^{n_{\text{u}}}$ describes the experimental setup (see explanation below).

The state *x*(*t*) is the current condition of the system, whereas the flux *v*(*x*, *θ*, *u*) determines the change of the state with time. The flux *v_j_*(*x*, *θ*, *u*) corresponds to the frequency with which reaction *R_j _*takes place. If mass action kinetics [1] are assumed, we obtain

$$v_{j}\left(x,\theta ,u\right)=\kappa_{j}\overset{n_{\text{x}}}{\underset{i=1}{\prod }}x_{i}^{s_{ij}^{\left(\text{r}\right)}},\;j=1,\ldots ,n_{\text{r}}.$$

In this case, the parameters $\theta ={\left(\kappa_{1},\ldots ,\kappa_{n_{\text{r}}}\right)}^{\text{T}}$ are reaction rate coefficients (e.g., affinities) and exactly one parameter is associated with each reaction. If more complex flux models are used, such as Michaelis-Menten or Hill-kinetics [1], several parameters can be associated with one reaction. A simple example is the enzymatic conversion of *X_i _*into *X_i∗_*by the enzyme $X_{i^{E}},X_{i}+X_{i^{E}}\to X_{i*}+X_{i^{E}}$, often described using the Michaelis-Menten kinetics,

$$v_{j}\left(x,\theta ,u\right)=\frac{\kappa_{j,\text{max}}x_{i}}{\kappa_{j}+x_{i}}.$$

In this case, two parameters are assigned to reaction *j*: *κ*_*j*,max _and *κ_j_*.

In a graph theoretical context, the time-dependent states *x_i_*(*t*) are attributes of the vertices and the time-dependent fluxes *v_j_*(*x*(*t*), *θ*, *u*(*t*)) as well as the fixed parameters *θ_j _*are attributes of the edges.

ODE-based modeling of BRN is flexible and allows for the description of many metabolic, signal transduction, and gene regulation processes. However, like most other modeling approaches it suffers from one major problem. Due to experimental constraints, the parameters *θ_j _*cannot be measured directly, but have to be estimated.

#### Bayesian parameter estimation

To estimate the parameters *θ_j_*, measurement data have to be collected. The measured quantities $y\left(t\right)\in {\mathbb{R}}_{+}^{n_{\text{y}}}$ (also called measurands, observables, or outputs),

(2) $$y_{k}=h_{k}\left(x,\theta \right),\;\;k=1,\ldots ,n_{\text{y}}\text{,}$$

are typically individual states (*h_k_*(*x*, *θ*) = *x_i_*), sums of states $\left(h_{k}\left(x\right)=x_{i_{\text{1}}}+x_{i_{\text{2}}}\right)$, or quantities that are proportional to one of the aforementioned ones. As the measurements are corrupted by noise, the available data are:

$$D={\left\{(y(t_{l}),t_{l})\right\}}_{l=1}^{n_{t}}\;\text{with}\;y_{k}(t_{l})=y_{k}(t_{l})+\varepsilon_{k}(t_{l}),$$

in which $t_{l}\in {\mathbb{R}}_{+},ȳ\left(t_{l}\right)\in {\mathbb{R}}_{+}^{n_{\text{y}}}$, and $\varepsilon \left(t_{l}\right)\in {\mathbb{R}}^{n_{\text{y}}}$ denote the time at which the measurement was performed, the noise-affected output, and the measurement noise, respectively.

In a graph context, the measured quantities $y_{k}$ represent an additional layer. This layer contains the different outputs, $h_{k}\left(x,\;\theta \right)$, as elements with the two time-dependent attributes: the simulated output trajectories $y_{k}\left(t;\;\theta \right)$ for particular parameter values; and the discrete measured quantities ${ȳ}_{k}\left(t_{l}\right)$. The measured quantities depend on one or more states (concentrations) in the network but do not interact with these components. Thus, the measured quantities represent sets of vertices of the chemical reaction network, where these sets contain all chemical species $X_{i}$ with $\frac{\partial }{\partial x_{i}}h_{k}\left(x,\theta \right)\neq 0$.

Measurement data are in general available for different experimental conditions. The experimental conditions are described using the function *u*(*t*), which might be time-dependent. The experiment description $u^{e}\left(t\right)$ of the *e*-th experiment can account for different interventions, e.g., silencing or over-expression of genes, stimuli, and medium changes. Thus, it alters the reactions rates. As $u^{e}\left(t\right)$ differs for the individual experiment, so do the time-dependent outputs $y^{e}\left(t\right)$, measured outputs ${ȳ}^{e}\left(t\right)$, states $x^{e}\left(t\right)$, and fluxes $v^{e}\left(x\left(t\right),\theta ,u^{e}\left(t\right)\right)$. Furthermore, the data used for parameter estimation are the union of the data obtained from the individual experiments, $D=\cup_{e}D^{e}$. As all statements hold for all experiments, in the following we skip the superscript *e *to simplify the notation. Given the data  D, the parameters are estimated. For this purpose, different methods can be employed. One commonly used method is Bayesian parameter estimation [4,8], which relies on Bayes' theorem,

(3) $$p\left(\theta |D\right)=\frac{p\left(D|\theta \right)p\left(\theta \right)}{p\left(D\right)}.$$

The expression on the right hand side of (3) provides the posterior probability $p\left(\theta |D\right)\in {\mathbb{R}}_{+}$ of a parameter vector *θ*, given the data  D. Here, the conditional probability to observe the data $p\left(D|\theta \right)\in {\mathbb{R}}_{+}$, the prior knowledge of the parameters $p\left(\theta \right)\in {\mathbb{R}}_{+}$, and the prior probability of the data $p\left(D\right)\in {\mathbb{R}}_{+}$ are taken into account. In the case of independent normally distributed measurement noise, $\varepsilon_{k}\left(t_{l}\right)\sim N\left(\varepsilon |0,\sigma_{k}^{2}\left(t_{l}\right)\right)$, the conditional probability becomes

$$p\left(D|\theta \right)=\overset{n_{\text{y}}}{\underset{k=1}{\prod }}\overset{n_{\text{t}}}{\underset{l=1}{\prod }}\frac{1}{\sqrt{2\pi }\sigma_{k}\left(t_{l}\right)}\text{exp}\left(-\frac{1}{2}{\left(\frac{{ȳ}_{k}\left(t_{l}\right)-{ȳ}_{k}\left(t_{l};\theta \right)}{\sigma_{k}\left(t_{l}\right)}\right)}^{2}\right),$$

in which $y_{k}\left(t_{l};\theta \right)=h_{k}\left(x\left(t_{l};\theta \right),\theta \right)$ is the simulated output of the model (1) and (2). The conditional probability and thus the posterior probability is large, if the distance between measurement and data is small. A high value of p(D|θ) indicates that the considered parameter vector *θ *is plausible and might be close to the true parameter of the biological process.

#### Uncertainties of parameters, fluxes, and states

In general, the parameters *θ_j _*cannot be determined precisely but remain uncertain. This uncertainty is encoded in the shape of the posterior probability p(θ|D). Large uncertainty is indicated by a broad distribution, while, e.g., dependencies between a subset of the parameters might result in a narrowing of distributions in certain subspaces or manifolds. As the number of unknown parameters *θ *is often large, *n_θ _*≫ 1, the analysis of p(θ|D) is challenging. To analyze the uncertainty, a sample ${\left\{\theta^{\left(\alpha \right)}\right\}}_{\alpha =1}^{n_{\text{s}}}$ is generated from p(θ|D), using Markov chain Monte Carlo (MCMC) sampling [9]. Associated with this parameter sample, we have a flux sample ${\left\{v^{\left(\alpha \right)}\left(t\right)\right\}}_{\alpha =1}^{n_{\text{s}}}$ and a state sample ${\left\{x^{\left(\alpha \right)}\left(t\right)\right\}}_{\alpha =1}^{n_{\text{s}}}$. The individual members of this sample are flux trajectories $v^{\left(\alpha \right)}\left(t\right)=v\left(x^{\left(\alpha \right)}\left(t\right),\theta^{\left(\alpha \right)},u\left(t\right)\right)$ and state trajectories $x^{\alpha }\left(t\right)$, respectively. These trajectories are obtained by simulating the model (1) for parameter $\theta^{\alpha }$. The samples ${\left\{\theta^{\left(\alpha \right)}\right\}}_{\alpha =1}^{n_{\text{s}}},{\left\{v^{\left(\alpha \right)}\left(t\right)\right\}}_{\alpha =1}^{n_{\text{s}}}$, and ${\left\{x^{\left(\alpha \right)}\left(t\right)\right\}}_{\alpha =1}^{n_{\text{s}}}$ carry the statistical properties of p(θ|D) as well as its image in flux and concentration space. Hence, the samples can be used to gain insight into the parameter and prediction uncertainties.

### Analysis goals: understanding uncertainty and process dynamics

Understanding the parameter and prediction uncertainties is crucial to ensure a good understanding of the model and its limitations, and to support the comparison of performed experiments as well as the selection of future experiments. Unfortunately, the in-depth analysis of model uncertainty is ambitious because it requires the analysis of hidden dependencies between the static and dynamic attributes of the model. While these dependencies could theoretically be detected algorithmically, the fact that the interesting features—the things we are looking for during the exploration phase—are not known a priori, complicates algorithmic searches in practice. Visualization in combination with human perception has proven to be more powerful for exploration tasks [10] than algorithmic approaches.

So far, mainly tables, scatter plots, and line plots of existing systems have been used by domain experts to investigate parameter, flux and state samples. Using such visualizations independently, it is not possible to obtain a detailed view of the distributions and, hence, it is hard to detect complex patterns within the data. In contrast, using linked visualizations for the analysis of individual attributes of the BRN model allows to achieve this analysis goal. In particular, exploration approaches, which allow the user to subsequently focus on different aspects of interest, are essential ingredients. These include the assessment of relatively high uncertainties and the identification of uncertainty hubs. Besides, the analysis of time dependence of outputs, fluxes, and states and their time-dependent uncertainties as well as localizing hubs involved in fast or slow process dynamics is of interest. Furthermore, it is necessary to characterize correlations between attributes, e.g., between parameters, fluxes, or states. Finally, the comparison of uncertain fluxes, states, and outputs between different experimental conditions is important to understand how particular aspects of the dynamics are altered.

### Related work

BRNs are usually displayed as node-link diagrams, where chemical species (vertices) are represented as nodes and reactions (directed edges) by links with arrow heads connecting the nodes. The vertices and edges of a network carry domain-specific attributes; here parameters, fluxes, and states. These can be mapped to visual attributes of nodes and links, such as their thickness, brightness, shape, or color [11]. While the structure of BRNs is static, attributes attached to vertices (states) and edges (fluxes) may be dynamic. There are three common approaches to integrate the evolution of multi-dimensional information into graph visualizations [12]: small multiples [13], animation, and complex glyphs, such as small charts embedded into the graph.

Although there is a large number of visualization tools for BRNs, only few of them support the visualization and the visual comparison of dynamic node attributes of experimental data collected under different experimental conditions. For the simultaneous visualization of gene regulatory networks and their states at different time points *Cerebral *[14], *Pathline *[15], *GENeVis *[16], *VANTED *[17] and the *Pathway Tools *software [18], can be used. *Cerebral *and *Pathline *exploit small multiple views of the graph or line charts of the time-series data, respectively. *GENeVis *and *VANTED *make use of small charts embedded into the vertices. All these visualization approaches perform well if the number of time points is small, however, they do not scale well. Furthermore, small multiples and small embedded charts do not allow for the comparison of the time series of different fluxes or different states for one experimental condition. To avoid some of these problems, in the *Pathway Tools *software the attribute dynamics are visualized using animation instead of a static representation. However, this does not facilitate the analysis of dynamic attributes across different experimental conditions.

Beyond the visual analysis of time dependencies of BRN attributes, also the attribute uncertainties have to be studied to assess the reliability of model predictions. Unfortunately, hardly any tool exists that can visualize the uncertainties associated with the graphs attributes. Commonly used tools like *COPASI *[19] and *CellDesigner *[20] support the simulation of BRN models and visualization of model predictions for a given parameter value, however, they do not provide visualizations of the prediction uncertainties. In addition, the available tools do neither support a visual exploration of the dynamics and uncertainties, nor link the information with the underlying graph structure of the BRN.

The visualization of uncertainties has been recognized as one of the key challenges in scientific visualization [21]. Therefore, in different scientific disciplines, e.g., flow field visualization and surface representations [22,23], much research has been carried out on uncertainty visualization. In contrast, little work has been done on visualizing the uncertainties of graph attributes. To study multiple static node attributes Cesario et al. [24] introduced a method which employs spatial layouts in combination with different linked views including parallel coordinates, scatter plots, comparative columns and bullseyes. To visualize structural uncertainties of graphs, Lee et al. [25] developed *CandidTree*. We are not aware of tools that visualize attribute uncertainties, quantified in terms of standard deviation, percentiles or other uncertainty measures, directly within the graph. This could by achieved by animation, adding glyphs or geometry to the rendered scene, modifying geometry, or addressing other human senses [26].

## Methods

We developed the visual analytics system *iVUN*, a JAVA based tool facilitating the interactive visualization of uncertain BRNs. *iVUN *has been developed in a participatory design process together with two target users. To assess the utility of the individual visualization approaches and multiple linked views of *iVUN*, a qualitative user study with 10 domain experts was performed. A more detailed description of the design process as well as the design and results of the qualitative user study can be found in our previous work on the uncertainty-aware visual analysis of BRNs [6].

### Overview about iVUN

**Figure 2:**
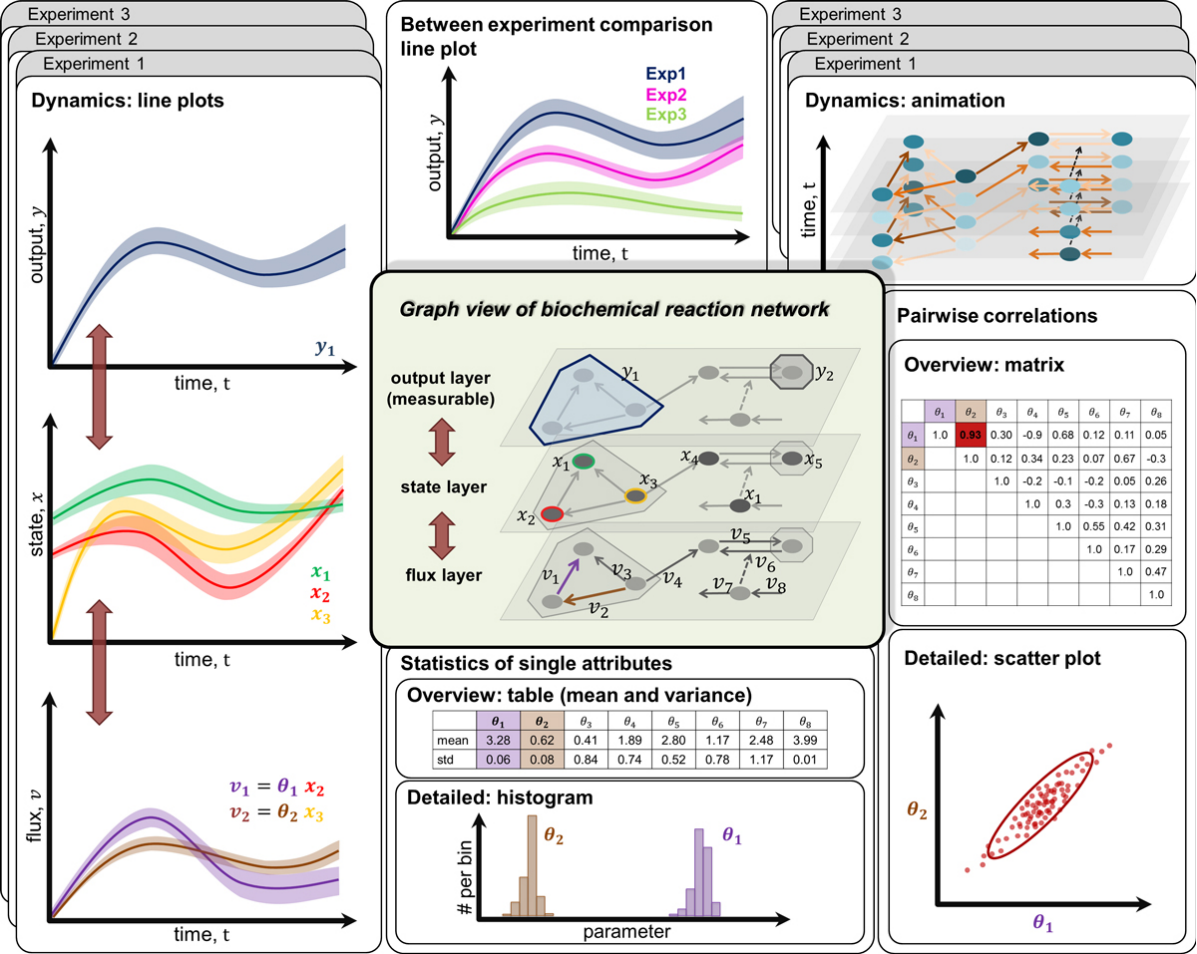
**Conceptual overview of *iVUN *functionality**. The central component of *iVUN *is the graph view of the BRN, with which all static and dynamic attributes are associated. From a modeling perspective, this graph has several layers. The dynamic attributes v , x , and y  are associated with the flux layer (links), state layer (nodes), and the output layer (a subset of nodes), respectively. These layers are connected, i.e., an output (e.g., $y_{1}$) is associated with a set of states (here, $y_{1}$ represents the sum of $x_{1}$, $x_{2}$, and $x_{3}$) and each state depends on one or more fluxes (here, $x_{1}$ depends on $v_{1}$ and $v_{2}$). Conversely, a flux *v*_*j *_influences one or more states $x_{i}$, which may be part of one or more outputs $y_{k}$. To obtain an overview of the attribute dynamics, *iVUN *can map the numerical value of attributes to the color of the respective components in the BRN and animate the time dependence (illustration: top right). In addition, line plots can be used to gain further insight into the dynamic behavior and to compare time series. *iVUN *supports within- experiment comparison (line plots: left) and between-experiment comparison (line plot: top center) of time series. Besides the analysis of dynamics, the analysis of statistics of single attributes (bottom center) and correlations (right) is supported at different levels of granularity. In this conceptual overview, colors are used to identify the dynamic attributes in the line plots as well as the static parameters in the statistic and correlation views with the corresponding components of the graph. In the actual visual analytics tool *iVUN*, correspondence of elements in the different views is shown by brushing and linking as well as labeling. In this way, color can be used for encoding of other values. Screenshot of *iVUN *in action can be found at www.vis.uni-stuttgart.de/iVUN.

*iVUN *imports the BRN from an xml file following the specifications SBML [27]. The BRN is visualized as a node-link diagram (graph view), where nodes represent species and links (arrows) represent reactions (see Figure 2: graph view).

As presented in the section *Computational Modeling of Biochemical Reaction Networks*, the BRNs have different attributes. MCMC sampling does not only provide a sample of the parameters ${\left\{\theta^{\left(\alpha \right)}\right\}}_{\alpha =1}^{n_{\text{s}}}$, but also of the outputs ${\left\{y^{\left(\alpha \right)}\left(t\right)\right\}}_{\alpha =1}^{n_{\text{s}}}$, the states ${\left\{x^{\left(\alpha \right)}\left(t\right)\right\}}_{\alpha =1}^{n_{\text{s}}}$, and the fluxes ${\left\{v^{\left(\alpha \right)}\left(t\right)\right\}}_{\alpha =1}^{n_{\text{s}}}$. The individual samples can be imported from text files in txt or csv format as described in the tutorial. The output, state, and flux samples depend on the experimental condition *u*^(*e*)^. This experimental context has to be conserved.

As mentioned in section *Bayesian Parameter Estimation*, outputs are typically individual state variables or sums of states and can therefore be assigned to one or more species and hence nodes in the graph. Thus, the outputs are visualized using convex hulls that surround the respective nodes.

To analyze the uncertainty of static parameters (*θ*) and experiment-dependent dynamic properties—utputs (*y*), states (*x*, also referred to as concentrations), and fluxes (*v*)—*iVUN *offers multiple linked views showing information at different levels of granularity. Brushing and linking techniques are used to connect views that share the same data attributes [28], i.e., to visually link the elements in the different views. A change in selection within one view by brushing directly results in the highlighting of the respective elements within all views. In particular, the elements are first highlighted by short flashing to attract the users attention before they stay highlighted.

An overview of the summary statistics of samples, like mean and standard deviation, can be obtained from the graph view by mapping them to visual attributes of the graph but also in a linked table view. Further linked views support the analysis of the distribution of values within samples, the dynamic change of samples as well as correlations between sample members or time courses (see Figure 2). In addition, *iVUN *supports the comparison of different experiments and hence sets of outputs, flux samples, and state samples.

In the following subsection, we present the visualization methods included in *iVUN*:

• Histograms and color mapping in the graph view and table views for the analysis of parameter uncertainties.

• Different types of line plots and animation of the graph view for the analysis of attribute dynamics.

• Scatter plots and correlation matrices for the analysis of attribute correlations.

• Combinations of different tools and the linking between them to explore complex, hidden dependencies.

The different visualization methods have been combined to meet the aforementioned analysis goals.

### Visualization of statistics of attribute samples

One analysis task is the localization of large uncertainties and uncertainty hubs in the BRN. To support this, statistical properties of the parameter sample ${\left\{\theta^{\left(\alpha \right)}\right\}}_{\alpha =1}^{n_{\text{s}}}$, the flux sample ${\left\{v^{\left(\alpha \right)}\left(t\right)\right\}}_{\alpha =1}^{n_{\text{s}}}$, and state sample ${\left\{x^{\left(\alpha \right)}\left(t\right)\right\}}_{\alpha =1}^{n_{\text{s}}}$ can be color-coded in the graph. *iVUN *supports the mapping of the mean and standard deviation of the samples to the color of links (for parameters and fluxes) and nodes (for states). As edges possess two attributes, the user can select whether the parameters or the fluxes are visualized.

For kinetic rate laws with several parameters, the links are split into the respective number of segments. Each segment is colored according to the statistical properties of one parameter: starting with the first parameter of the kinetic law at the starting point of the edge and ending with the last parameter of the kinetic law at the arrowhead. A disadvantage of this approach is the fact that the number of parameters as well as the link length can differ for different reactions. For that reason, the length of individual link segments is not necessarily uniform. Nevertheless, this approach enables the simultaneous assessment of the uncertainty of all parameters and avoids the switching between different parameters. Therefore, it is possible to perceive whether all parameters of a reaction are (un-)certain or whether the uncertainties differ for the different parameters of the reaction. For an individual mapping of mean values or standard deviations, *iVUN *offers different colormaps created with *ColorBrewer *[29]. Based on these color maps, users can identify relatively low and high values. For the simultaneous visualization of mean and standard deviation, bivariate multi-hue colormaps are provided. If several experiments are imported with different output, state, and flux samples, nodes and links are colored based on the statistical attributes of the currently selected experiment.

In addition to the graph-based visualization, means and standard deviations of the parameters are summarized in a table view. This table is linked to the graph view. All reactions associated with the parameter selected in the table view are highlighted in the graph view. Furthermore, when selecting a reaction, all associated parameters are highlighted in the table view. The cells of the table are colored using the same color maps as used for the links to visualize mean values and standard deviations. Hence, the lowest and highest mean values and standard deviations for the parameters of the system can be identified at a glance. This supports the assessment of relatively low and high uncertainties.

For the detailed investigation of the parameter distributions, *iVUN *provides a histogram view. This view includes the histograms for all selected parameters (see Figure 2: statistics). The histograms of the different parameters are comparable as they are computed with the same bin width.

### Visualization of the attribute dynamics

BRNs are dynamical systems and therefore fluxes, states, and outputs are time-dependent. These time-dependent attributes have to be compared within and across experiments to understand the systems' behavior. In addition, outputs, states, and fluxes are intertwined: the fluxes determine the states, and the states determine the outputs. This hierarchical structure of BRNs is exploited by *iVUN*, which is why we study the flux, the state, and the output layers. *iVUN *incorporates animation and linked line plots to visualize attribute dynamics (see Figure 2: dynamics). The animation allows the user to obtain an overview, e.g., to detect drastic changes, and to identify hubs of similar dynamics [6]. Thereby, the time-dependent means or standard deviations of the sample ${\left\{v^{\left(\alpha \right)}\left(t\right)\right\}}_{\alpha =1}^{n_{\text{s}}}\left({\left\{x^{\left(\alpha \right)}\left(t\right)\right\}}_{\alpha =1}^{n_{\text{s}}}\right)$ are mapped to the color of the respective link (node). iVUN supports a navigation through time either stepwise using the forward or backward button or rapidly with the help of a slider. Continuous animation is obtained by keeping these buttons pressed.

Animation poses a natural way to convey dynamic data, whereas at the same time its effectiveness is limited due to perceptual and cognitive limitations in the processing of changing visual presentations [30]. To improve the perception during the continuous animation, drastic changes of the mean or standard deviation of the samples are automatically detected and respective links (nodes) are briefly highlighted within the graph view [31]. While this yields some improvement, it still does not allow for a detailed analysis and a quantitative comparison of all time series for fluxes or states. Therefore, we visualize the dynamic behavior of outputs ${\left\{x^{\left(\alpha \right)}\left(t\right)\right\}}_{\alpha =1}^{n_{\text{s}}}$, states ${\left\{x^{\left(\alpha \right)}\left(t\right)\right\}}_{\alpha =1}^{n_{\text{s}}}$, and fluxes ${\left\{v^{\left(\alpha \right)}\left(t\right)\right\}}_{\alpha =1}^{n_{\text{s}}}$ in separate line plots. Each line within the respective plot represents the time-dependent median of one output, one state, or one flux. To visualize the uncertainties, we frame the lines corresponding to the medians by a semitransparent area those boundaries are the time-dependent percentiles of the respective sample (by default the 5th-percentile, *P*_5_, and the 95th-percentile, *P*_95_). The use of the median and the percentile intervals allows for the study of asymmetries in the distribution.

For the outputs, median and percentile intervals of the simulated trajectories as well as the measured data are included in the line plot. As the measured values ${ȳ}_{k}\left(t_{l}\right)$ are only available at discrete time points, they are depicted as dots. The colors of simulated trajectories *y_k_*(*t*) and measured points ${ȳ}_{k}\left(t_{l}\right)$ are identical. To visually link the outputs in the line plot with the respective set of nodes (convex hull) in the graph view, the same color is also used for the convex hull. One essential feature of *iVUN *that supports the analysis of complex networks is that line plots and graph view are directly linked. A selection in a line plot results in the highlighting of the corresponding nodes or links in the graph view, and vice versa. As outputs are in general weighted sums of states and hence associated with more than one species, several species (nodes) are highlighted. Conversely, the selection of a species that belongs to several outputs, results in the highlighting of all these outputs in the line plot.

As the number of species and reactions increases for more complex BRNs, the line plots for states, outputs, and fluxes become cluttered. Furthermore, if the number of lines is greater 10, the corresponding colors become difficult to distinguish. Therefore, instead of just highlighting selected elements to focus on fluxes, states, or outputs of interest, lines can be faded out using check boxes.

For all dynamic attributes, two types of line plots are available: a line plot to compare time series of different attributes for the currently selected experiment (see Figure 2: within-experiment comparison line plots); and a line plot to compare the currently selected attributes for all experimental conditions (see Figure 2: between-experiment comparison line plots). With these views, users can investigate and compare uncertainties and dynamics under different experimental conditions.

### Visualization of correlations between attributes

The uncertainty of one attribute often comes along with uncertainties in some other attributes. Furthermore, the values of two attributes, e.g., two parameters in ${\left\{\theta^{\left(\alpha \right)}\right\}}_{\alpha =1}^{n_{\text{s}}}$, might be correlated. Identifying these interdependencies is essential to understand the behavior of the system. Therefore, *iVUN *supports the identification of correlations between uncertain attributes.

**Figure 3:**
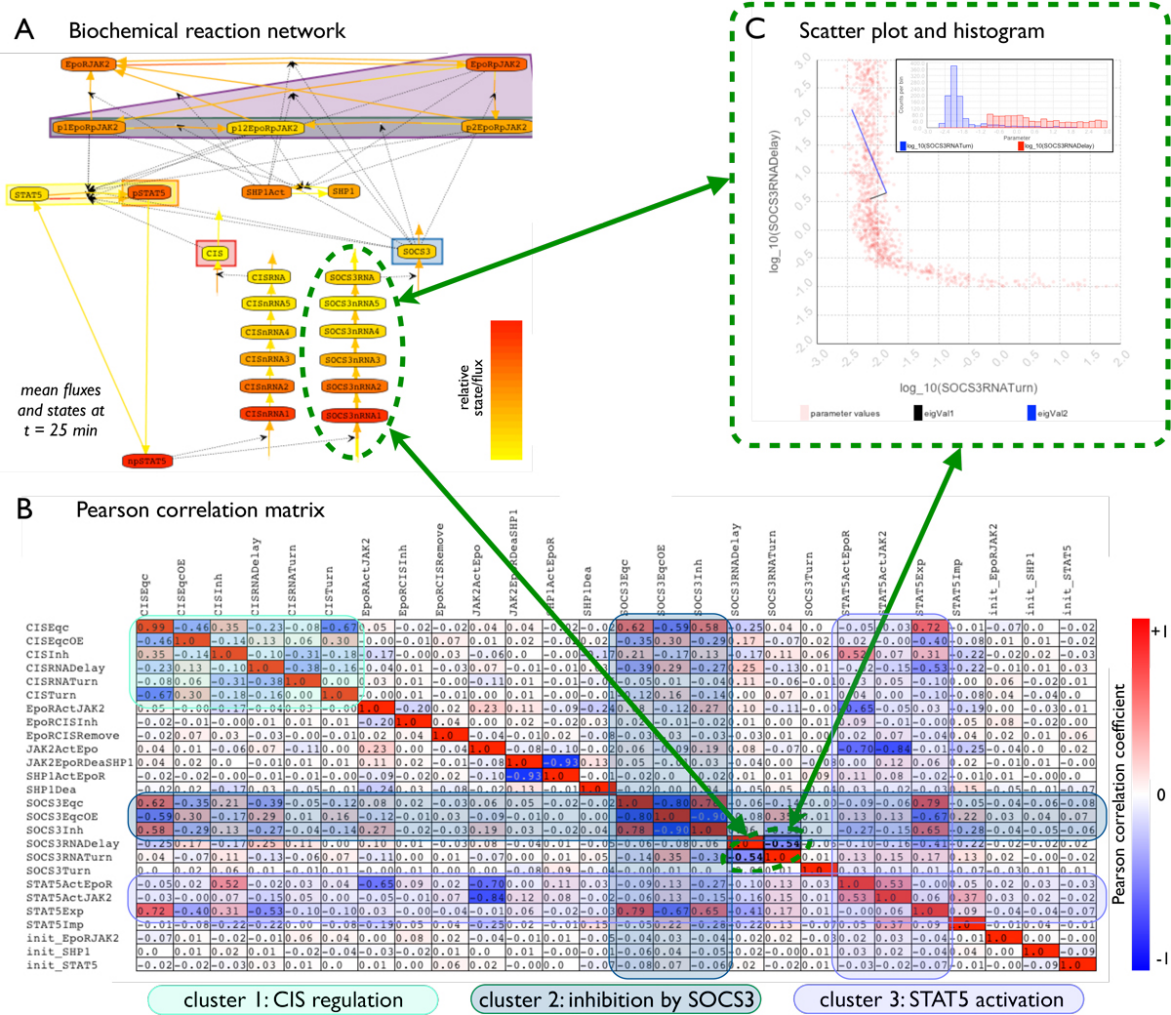
**Analysis of parameter correlations in the JAK2/STAT5 signaling pathway**. By linking the graph view (A) with the Pearson correlation matrix (B), scatter plots and histograms (C), our visual analytics systems *iVUN *allowed for a step-by-step exploration of the MCMC sample obtained for the JAK2/STAT5 signaling pathway [34]. The graph view (A) shows the reactions (links), states (nodes), and outputs (subsets of nodes surrounded by a semitransparent area). The sample mean of fluxes and states is mapped to the color of links and nodes respectively. The Pearson correlation matrix (B) facilitates the efficient search for pairs of strongly correlated parameters. The selection of individual parameter pairs in the Pearson correlation matrix followed by the automatic highlighting of associated edges in the graph view (A) allows for the identification of these parameters in the BRN. In the JAK2/STAT5 signaling pathway, pairs of strongly correlated parameters in general influence similar states and outputs of the network. In addition, there are some parameters that alter several reaction fluxes, e.g., 'SOC3Inh', and which therefore correlate with many parameters. This yields clusters of strong parameter-wise correlations, which are recognizable in the matrix view. Beyond the analysis of linear correlations using the Pearson correlation matrix, scatter plots (C) reveal nonlinear correlation structures as shown for 'SOCS3RNADelay' and 'SOCS3RNATurn'. The parameter sample for the JAK2/STAT5 signaling pathway shows only few strongly nonlinear correlations, although histograms (C) reveal that, e.g., the distribution for the parameter 'SOCS3RNATurn' is bimodal.

Two different matrix views are available that can be used to investigate dependencies of or correlation between different dimensions of the parameter sample ${\left\{\theta^{\left(\alpha \right)}\right\}}_{\alpha =1}^{n_{\text{s}}}$: an eigenvalue-ratio-matrix and a correlation matrix. The former is based on principal component analysis (PCA), whereas the latter provides the Pearson's correlation coefficients for all pairwise combinations of parameters. For dynamic attributes, the correlation matrix displays the pairwise Pearson's correlation coefficients between the sample members of the currently selected time point or between time courses of either mean values or standard deviations. The cells within this matrix include the numerical values and are colored with respect to the sign and absolute value of the ratio (see Figure 3B).

Similar to the line plots, also the matrix views are linked to the graph view. If a cell within a matrix is selected, the two respective elements within the graph as well as the respective columns within the table and lines within the line plots are highlighted. Vice versa, if the user selects a set of elements (*θ*, *v*, or *x*) within the graph, all pairwise combinations and hence respective cells within the matrix views are highlighted.

While correlation matrices allow for an overview of occurring correlations, scatter plots can be used to gain further insights into the type of correlation and the properties of the distribution (see Figure 2: correlations and Figure 3B and 3C). To facilitate the exploration, the scatter plot of two elements can be obtained by selecting either the respective elements in the graph or the respective cell in the matrix. For fluxes and states, the sample for the currently selected time point *t_k _*is visualized within the scatter plot.

For more details on the visualization tools and the implementation, we refer to the documentation of *iVUN *(http://www.vis.uni-stuttgart.de/iVUN) and our previous publication [6].

## Results and discussion

### JAK2/STAT5 signaling pathway

For this case study, we consider the Epo-induced JAK2/STAT5 signaling pathway. The hormone Erythropoietin (Epo) regulates the production of red blood cells. Initially, Epo binds to the Epo-receptor inducing a rapid phosphorylation of JAK2. Phosphorylated JAK2 activates the transcription factor STAT5 by phosphorylation. Phosphorylated STAT5, pSTAT5, can be translocated from the cytosol to the nucleus where it induces the transcription of CIS and SOCS, two inhibitors of JAK2 and STAT5 phosphorylation. The dual feedback loop established by CIS and SOCS adjusts STAT5 phosphorylation levels over the entire range of Epo concentrations. This is essential as *in vivo *a broad dynamic range of Epo concentration is observed [32] and STAT5 influences the cell fate. It has been shown that pSTAT5 promotes the survival of erythroid progenitor cells [33].

In the following, we consider the ODE model of the Epo-induced JAK2/STAT5 signaling pathway introduced by Bachmann et al. [33] (see Figure 3A). This model describes the time-dependent concentrations of 25 chemical species under 24 experimental conditions. It is highly nonlinear and possesses 113 unknown parameters. Due to the high dimensionality of the parameter space, Bayesian parameter estimation for this problem is challenging. Recently, a novel adaptive hierarchical MCMC sampling scheme suited for this high-dimension problem was proposed [34]. Using the resulting MCMC sample, the uncertainty of the individual parameters and the prediction uncertainty of the concentration of nuclear pSTAT5 and SOCS3 was evaluated using classical approaches. Here, we use the same MCMC sample to study the uncertainties and correlations of parameters, fluxes, states, and outputs in more detail. To simplify the analysis slightly, we focus on the 27 dynamic parameters and initial conditions that influence the biochemical process. The 86 nuisance parameters, which are necessary to compare the model with the experimental data, are not analyzed in detail because this does not promise additional biological insight.

Note that the outputs of the model are log-transforms of concentrations and can thus be negative. The log-transformation is necessary to account for the noise distributions observed in biological systems. As the logarithmic scale is a natural choices for the strictly positive physical quantities [35], such as reaction rates, we analyze the logarithmic values of the parameters, log_10_(*θ*), and their statistics throughout this section.

### Analysis of parameter uncertainties and correlations

To improve our understanding of inter-dependencies and correlations of the unknown parameters, we investigated the graph view of the BRN, the Pearson correlation matrix, and the scatter plots. The Pearson correlation matrix provides a rough overview of the correlation structure, whereas the linking to the graph view—reactions associated to selected parameter pairs are highlighted—allows us to locate the parameters in the BRN. Using this linking, we can analyze visually whether strongly correlated parameters are in proximity to each other—as one would expect. This is an essential advantage of the linking as the model equations are complex and the parameter names are not fully intuitive.

Furthermore, for this system several states and reaction rates have been scaled to circumvent structural non-identifiability. This increases the model complexity and renders the identification of parameters in the BRN, without the need to study the model equations, a powerful feature.

For the JAK2/STAT5 pathway, our analysis using the graph view in combination with the correlation matrices and scatter plots indeed revealed that most of the strongly correlated parameters are in close proximity to each other, influencing, e.g., the in- and outflux in one state (positive correlation) or two influxes (negative correlation). In Figure 3(A) one such parameter pair is highlighted. Using *iVUN*, we could easily detect several existing "correlation clusters" that are a result of the strong, localized correlations. Figure 3B depicts these correlation clusters which determine: the modulation of STAT5 phosphorylation via CIS (cluster 1); the inhibition of JAK2 phosphorylation by SOCS3 (cluster 2); and the STAT5 activation by different forms of the receptor (cluster 3). In addition to these correlation clusters, there are some parameters, e.g., 'SOC3Inh', which influence many reaction rates and are therefore correlated with many reaction rates.

Beyond the analysis of the Pearson correlation matrix, we employed scatter plots and histograms to assess a few interesting parameter combinations, e.g., parameters that belong to different mechanisms. One interesting example is the pair of 'SOCS3RNADelay' and 'SOCS3RNATurn', which shows medium negative correlation. The scatter plot reveals that the dependence between these parameters is highly nonlinear, and the histogram shows that the distribution of 'SOCS3RNATurn' is indeed bimodal. This has also been observed in [34] and analyzed using support vector machines, but our visual exploration requires less time and is in this regard advantageous.

### Analysis of output, state and flux dynamics and uncertainties

In most systems biology projects, the prediction of the response of a process, e.g., a signaling pathway, to altered experimental conditions is of primary interest. This requires the analysis of the dynamics attributes: outputs, states, and fluxes. The uncertainties of these three time-dependent quantities are expected to be significantly different. As the outputs have been measured at different discrete points, the uncertainty in the simulated output trajectories should be small. In contrast, states and fluxes are not directly measured but merely constrained by their relation to the output. Since one output is in general a weighted sum of several states, and only the sum of these states has to agree with the measured data, we expect that the uncertainty in the state trajectories is larger than in the output trajectories. Accordingly, the combination of several fluxes determines the time-evolution of the states, which can result in a further increase of the uncertainty.

**Figure 4:**
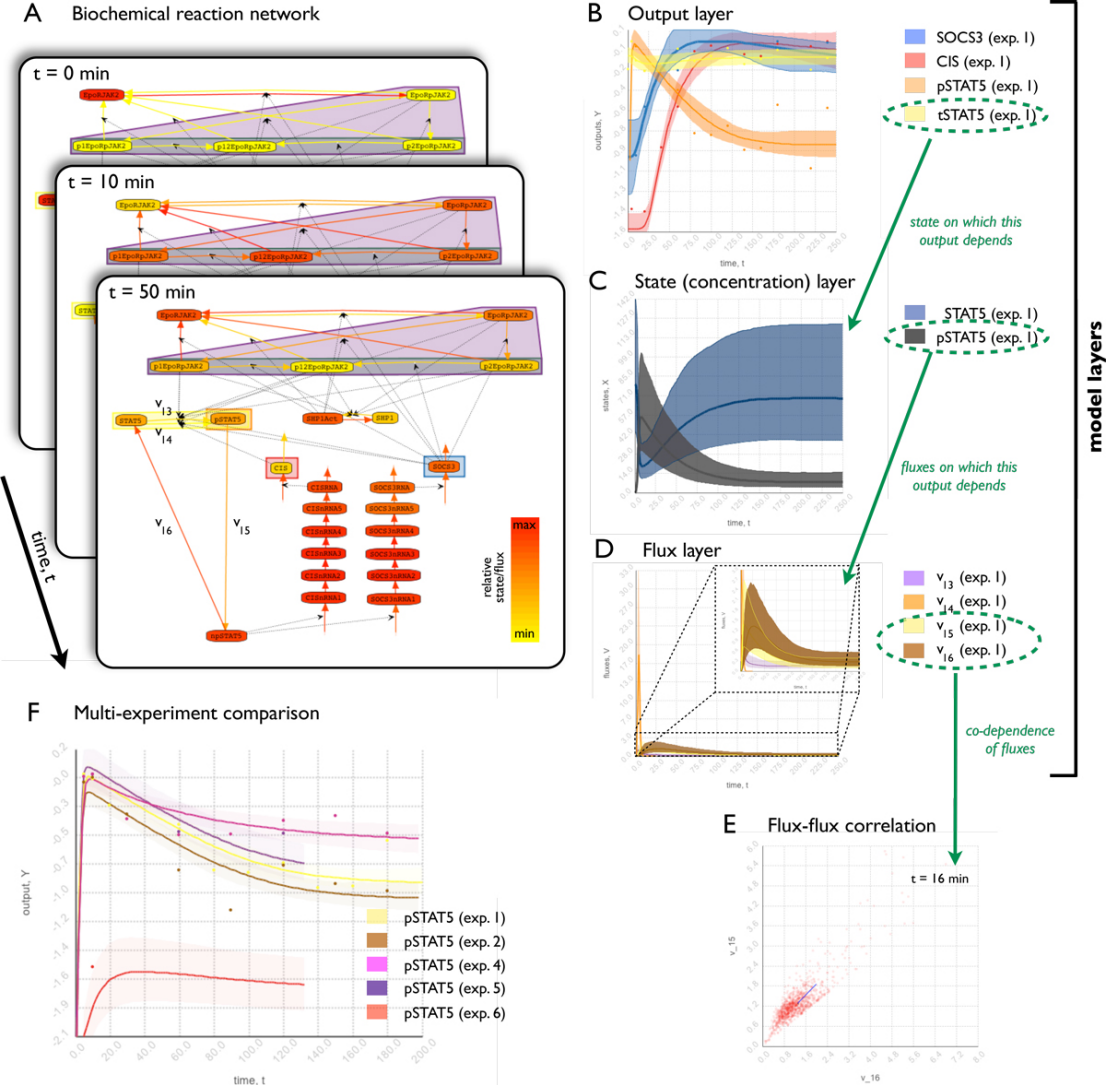
**Hierarchical analysis of the uncertain dynamics of the JAK2/STAT5 signaling pathway**. We made use of the linking between the graph view and the line plots, as well as the linking between different line plots, to perform a hierarchical analysis of the dynamic attributes of the JAK2/STAT5 signaling pathway (A). We studied the three layers of the BRN: the output layer (B), which includes the measured quantities; the state layer (C), which includes the states of the different chemical species; and the flux layer (D), which uniquely describes the changes in the states and the dynamics of outputs. The stack of graph views (A) illustrates the animation of the graph, where the states and fluxes at a particular time point are mapped to the color of the nodes and links respectively. The line plots (B)-(E) depict the median (full line) and the 90% Bayesian confidence interval (semitransparent area). Starting from the animation, the hierarchical analysis reveals that the output trajectories (B), which are fitted to the experimental data (dots), are the most well determined properties of the systems. The uncertainties in the states (C) that determine a certain output are in general much larger. Furthermore, different combinations of fluxes can yield roughly the same state trajectories, which in general results in a further increase of the uncertainties in the fluxes (D). However, for the JAK2/STAT5 signaling pathway this increase in uncertainty cannot be observed, probably, because the fluxes are constrained by molecular conservation laws. Variations in one flux must be compensated by another flux, resulting in significant flux-flux correlation (E). Complementary to the analysis of individual experimental data, the comparison across experiments provides information about the relative importance and role of certain mechanisms.

To assess these hypotheses, we analyzed the different layers of the JAK2/STAT5 pathway using a zooming approach. Starting with the animated graph view (Figure 4A) to gain an overview of the attribute dynamics, *iVUN *allows a step-by-step exploration of the output, state, and flux layers. Therefore, the corresponding line plots are linked. The selection of an output trajectory in the output line plot (Figure 4B) results in highlighting of the corresponding state trajectories in the state line plot (Figure 4C). Similarly, states and flux trajectories are linked (Figure 4D). Finally, the scatter plot of the flux can be depicted for different time points.

Using this zooming approach, we gained the impression that for the JAK2/STAT5 pathway the output uncertainty is indeed small compared to the uncertainty in the state trajectories. However, the state uncertainty appeared to be compatible with the flux uncertainty. This has also been confirmed using a numerical evaluation. We argue that the uncertainties of the fluxes are not larger than the uncertainties of the states, as most biological species are conserved. This provides an additional restriction on the fluxes. Furthermore, the highest node degree is 5 and most nodes have degrees 2 or 3. This small node degree limits the uncertainty of the fluxes further and results in significant correlations between fluxes, as illustrated in Figure 4E.

To compare the dynamics observed for different experimental conditions, *iVUN *allows for a "between-experiment comparison" of measurement data and simulation results using line plots. The line plots can directly reveal differences in: concentrations, slower/faster dynamics, peak concentrations and timing. In combination with the 90% confidence interval, we can furthermore conclude that, e.g., the pSTAT5 concentration varies significantly for different experimental conditions (Figure 4F).

A detailed description of how all plots for the case study were obtained can be found on the *iVUN *web page: http://www.vis.uni-stuttgart.de/iVUN.

## Conclusions

In this manuscript, we introduced the visual analytics system *iVUN*. *iVUN *has been devised to allow for the analysis of static and dynamic properties of BRNs described by ODEs. In many projects, the amount of available data is limited and the data are affected by noise. Therefore, the parameters, fluxes, states, and outputs of BRNs are not fully determined. A rigorous analysis requires the consideration of the limitation of the model. Therefore, *iVUN *supports an uncertainty-aware visualization by providing visualizations to study the statistics of uncertain parameters and confidence intervals for uncertain model predictions. These statistics are extracted from MCMC samples of parameters and the associated flux, state, and output trajectories.

The visualization of model uncertainties is one of two key advantages of *iVUN *compared to existing tools. The second key advantage is the linking of many different views (see Figure 2). *iVUN *provides a graph view to visualize the BRN, as well as secondary views depicting the time-dependence of samples (line plots), correlations between samples (scatter plot, correlation matrix), and statistics of samples (tables containing mean and variances). As the selection of elements in the secondary plots results in highlighting of the respective elements in the graph view, the user can explore the model properties without going forward and backward between plots and model equations. This allows for an improved understanding and a faster perception of the properties. A user study proved that a variety of questions can be answered using *iVUN *while accounting for the preferences of the individual users [6].

As illustrated in the case study of Epo-induced JAK2/STAT5 signaling, also new insight can be gained using *iVUN*. Concerning the parameters, one finding is that strong pairwise correlations occur mainly between parameters which are in close proximity to each other in the graph. With respect to output, state, and flux uncertainties, we could improve the understanding of uncertainty propagation across the layers in the presence of conservation relations.

Furthermore, due to its extensions compared to the previous version [6], *iVUN *allows for a detailed analysis of the agreement of model and data, as well as for the comparison of dynamic properties across different experimental conditions.

Beyond the analysis of BRNs governed by ODEs, *iVUN *is also capable of supporting the analysis of stochastic dynamics. *iVUN *merely requires a representative set of trajectories to evaluate the statistics. This set can also originate from a stochastic description of the BRN using, e.g., stochastic differential equation [36] or continuous time Markov processes [37], or a combination of parameter uncertainties and stochastic effects. Furthermore, besides sample-based Bayesian confidence intervals also other types of confidence intervals can be considered, e.g., profile likelihoods based confidence intervals [38,39].

In the future, we plan to extend the capabilities of *iVUN *even further toward a comprehensive analysis tool for BRNs. We want to allow for the abstraction of signaling pathways by aggregating subnetworks. To this end, the users will be enabled to define supernodes that represent these subnetworks. This is crucial as models become more and more complex. In addition to this coarsening, we want to allow for the assignment of detailed meta-information, e.g., references to the different species and reactions. Furthermore, additional views are planned for the assessment of high-dimensional dependencies, e.g., parallel coordinate plots, and we are awaiting the feedback of additional users. In summary, this paper introduced the software tool *iVUN *which has been devised for the study of graphs with uncertain, dynamic attributes. Using a model of the Epo-induced JAK2/STAT5 signaling pathway, the advantages of visual analytic approaches for the analysis of BRNs has been demonstrated on a real-world problem. While the tool is certainly not limited to this application, there is a particular need for uncertainty-aware visualizations. To inspire other researches to work on this problem and to test their methods, we propose the Epo-induced JAK2/STAT5 signaling pathway as a benchmark problem and provide all data at http://www.vis.uni-stuttgart.de/iVUN.

## Availability and requirements

*iVUN *is implemented in the Java™ programming language. For the network visualization, the Java Universal Network/Graph Framework Version 2.0.1 [40] is used. As basis for the diverse plots, including line plots, histograms and scatter plots, we use the Java library JMathTools [41]. The software and detailed documentation are available at www.vis.uni-stuttgart.de/iVUN. Furthermore, this web page provides all data for the case study and a description of how the plots shown in 'Results and Discussion' were obtained.

## Competing interests

The authors declare that they have no competing interests.

## Authors' contributions

CV, JH, AK, and DW devised and CV developed the visual analytics system *iVUN*. JH, SH, and AR analyzed the JAK2/STAT5 signaling pathway. SH, AR, JH, JT, and FT performed to the Bayesian parameter estimation for the JAK2/STAT5 signaling pathway. All authors wrote, read, and approved the final manuscript.

## Declarations

This publication was funded by the IEEE Symposium on Biological Data Visualization (BioVis) as a supplement of highlights.

The articles in this supplement have undergone the journal's standard peer review process for supplements. The Supplement Editors declare that they have no competing interests.

This article has been published as part of *BMC Bioinformatics *Volume 14 Supplement 19, 2013: Highlights from the 2nd IEEE Symposium on Biological Data Visualization. The full contents of the supplement are available online at http://www.biomedcentral.com/bmcbioinformatics/supplements/14/S19.
